# Reversing ADP-ribosylation

**DOI:** 10.7554/eLife.29942

**Published:** 2017-08-10

**Authors:** Giuliana Katharina Moeller, Gyula Timinszky

**Affiliations:** 1 Department of Physiological ChemistryLudwig-Maximilians-University of MunichMunichGermany; 2 Institute of GeneticsBiological Research Center of the Hungarian Academy of SciencesSzegedHungary

**Keywords:** PARP, ADP-ribosylation, ARH3, ADP-ribose, macrodomain, Human

## Abstract

The modification of serines by molecules of ADP-ribose plays an important role in signaling that the DNA in a cell has been damaged and needs to be repaired.

**Related research article** Fontana P, Bonfiglio JJ, Palazzo L, Bartlett E, Matic I, Ahel I. 2017. Serine ADP-ribosylation reversal by the hydrolase ARH3. *eLife* **6**:e28533. doi: 10.7554/eLife.28533

Cells rapidly react to stimuli in their environment by making modifications to proteins that change the way those proteins interact with other molecules (Mann and Jensen, 2003). Once a stimulus has stopped, these 'post-translational modifications' are usually reversed and the cell’s life goes back to normal. For example, when a cell suffers damage to its DNA, the addition of a molecule called ADP-ribose – a process that is known as ADP-ribosylation – to certain proteins sends a signal that leads to the damage being repaired; drugs that inhibit the addition of ADP-ribose are also used in cancer therapy (see Li and Yu, 2015 for a review).

It was discovered in the 1960s that specialized enzymes called PARPs can add one or more units of ADP-ribose (ADPr) to specific amino acids within proteins. Over the decades, it became clear that these enzymes are involved in a wide range of cellular processes, including DNA repair, transcription, chromatin regulation and cell death. The first target sites for ADP-ribosylation to be identified were mostly glutamates, aspartates and lysines, and the enzymes responsible for the removal of the ADPr units were also established (Figure 1)(Barkauskaite et al., 2013).

**Figure 1.**
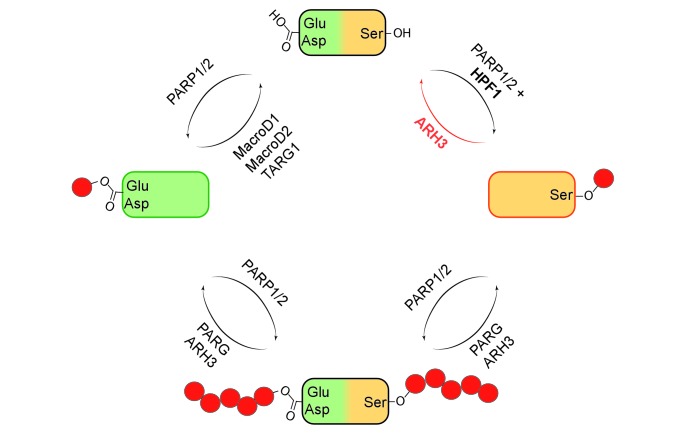
Mono- and poly(ADP-ribosyl)ation and their reversal. When a protein (top) undergoes mono(ADP-ribosyl)ation the ADP-ribose (red circle) can be added to a glutamate (Glu) or aspartate (Asp; left) or a serine (Ser; right). It is also possible for multiple units of ADP-ribose to be added to a protein at a given target site in a process known as poly(ADP-ribosyl)ation (bottom). The enzymes PARP1 and PARP2 are involved in ADP-ribosylation of both Glu/Asp and Ser, with a protein called HPF1 acting as a cofactor in the mono(ADP-ribosyl)ation of Ser. The enzymes involved in the reversal of both mono- and poly(ADP-ribosyl)ation are shown. Fontana et al. have shown that ARH3 is exclusively responsible for reversing the mono(ADP-ribosyl)ation of Ser, and that it is also involved (with PARG) in reversing the poly(ADP-ribosyl)ation of Ser.

More recently, it was shown that serines can be target sites for ADP-ribosylation, and that many of the proteins that contain such target sites have important roles in DNA damage repair (Bilan et al., 2017; Bonfiglio et al., 2017; Leidecker et al., 2016; Gibbs-Seymour et al., 2016). However, nothing was known about the enzymes or mechanisms responsible for the removal of the ADPr units from the serines. Now, in eLife, Ivan Ahel of the University of Oxford, Ivan Matic of the Max Planck Institute for Biology of Ageing in Cologne and co-workers – including Pietro Fontana, Juan José Bonfiglio and Luca Palazzo as joint first authors, along with Edward Bartlett – provide new insight into these matters (Fontana et al., 2017).

Using biochemical approaches and a technique called mass spectrometry, Fontana et al. screened a number of proteins that are known to bind to ADPr to find out if they could remove ADPr units that had been added to serines. They discovered that an enzyme called ARH3 could remove ADPr from serine on histone proteins (Figure 1). Previous research has shown that ARH3 and PARG work in similar ways. Both enzymes are able to break the ribose bonds that hold chains of ADPr units together, but ARH3 hydrolyses the chains less efficiently than PARG and also has a different structure (Mueller-Dieckmann et al., 2006; Oka et al., 2006). Fontana et al. discovered that unlike PARG, ARH3 was able to cleave both single ADPr units and chains of ADPr on histones and other proteins.

Since mass spectrometry is a rather expensive and laborious technique, Fontana et al. also used ARH3 in combination with western blotting – a basic technique to detect specific proteins or protein modifications – to track ADP-ribosylation on serines. These experiments confirmed the findings obtained with mass spectrometry, and proved that histone proteins are primarily – if not exclusively – modified on serine. Future studies could build on these findings and use ARH3 as a tool to detect the ADP-ribosylation of serines in proteins.

Despite these new insights, many outstanding questions remain. For example, how does adding ADPr to serine affect the role of a protein? And what happens when two neighboring amino acids experience post-translational modifications? A widely studied post-translational modification that regulates gene expression involves the methylation or acetylation of two lysines (K9 and K27) in histone three (Saksouk et al., 2015). However, these lysines are followed by a serine, which could undergo its own post-translation modification (which could be phosphorylation or ADP-ribosylation). Would these modifications influence each other? Probably, yes. This complex interplay may have far reaching consequences in the regulation of gene expression, and may play an important role in many diseases that depend on ADP-ribosylation pathways.
